# CRISPR-Cas9-mediated knockout of *SPRY2* in human hepatocytes leads to increased glucose uptake and lipid droplet accumulation

**DOI:** 10.1186/s12902-019-0442-8

**Published:** 2019-10-29

**Authors:** Naomi L. Cook, Milos Pjanic, Andrew G. Emmerich, Abhiram S. Rao, Susanne Hetty, Joshua W. Knowles, Thomas Quertermous, Casimiro Castillejo-López, Erik Ingelsson

**Affiliations:** 1 Molecular Epidemiology and Science for Life Laboratory, Department of Medical Sciences, Uppsala University, Uppsala, Sweden; 2 Department of Medicine, Division of Cardiovascular Medicine, Stanford University School of Medicine, Stanford, CA USA; 3 Molecular Systems Biology, Department of Cell and Molecular Biology, Uppsala University, Uppsala, Sweden; 4 Department of Bioengineering, Stanford University, Stanford, CA USA; 5 Stanford Cardiovascular Institute, Stanford University, Stanford, CA USA; 6 Stanford Diabetes Research Center, Stanford University, Stanford, CA USA; 7 Department of Immunology, Genetics and Pathology and Science for Life Laboratory, Uppsala University, Uppsala, Sweden

**Keywords:** SPRY2, Obesity, Type 2 diabetes mellitus, Hepatocytes, Glucose metabolism, Lipid metabolism, CRISPR-Cas9

## Abstract

**Background:**

The prevalence of obesity and its comorbidities, including type 2 diabetes mellitus (T2DM), is dramatically increasing throughout the world; however, the underlying aetiology is incompletely understood. Genome-wide association studies (GWAS) have identified hundreds of genec susceptibility loci for obesity and T2DM, although the causal genes and mechanisms are largely unknown. *SPRY2* is a candidate gene identified in GWAS of body fat percentage and T2DM, and has recently been linked to insulin production in pancreatic β-cells. In the present study, we aimed to further understand *SPRY2* via functional characterisation in HepG2 cells, an in vitro model of human hepatocytes widely used to investigate T2DM and insulin resistance.

**Methods:**

CRISPR-Cas9 genome editing was used to target *SPRY2* in HepG2 cells, and the functional consequences of *SPRY2* knockout (KO) and overexpression subsequently assessed using glucose uptake and lipid droplet assays, measurement of protein kinase phosphorylation and RNA sequencing.

**Results:**

The major functional consequence of *SPRY2* KO was a significant increase in glucose uptake, along with elevated lipid droplet accumulation. These changes were attenuated, but not reversed, in cells overexpressing *SPRY2*. Phosphorylation of protein kinases across key signalling pathways (including Akt and mitogen activated protein kinases) was not altered after *SPRY2* KO. Transcriptome profiling in *SPRY2* KO and mock (control) cells revealed a number of differentially expressed genes related to cholesterol biosynthesis, cell cycle regulation and cellular signalling pathways. Phospholipase A2 group IIA (*PLA2G2A*) mRNA level was subsequently validated as significantly upregulated following *SPRY2* KO, highlighting this as a potential mediator downstream of *SPRY2*.

**Conclusion:**

These findings suggest a role for *SPRY2* in glucose and lipid metabolism in hepatocytes and contribute to clarifying the function of this gene in the context of metabolic diseases.

## Background

The worldwide prevalence of obesity has markedly increased since 1980, with more than 609 million individuals classified as obese in 2015 [1]. Obesity (defined as a body mass index (BMI) of 30 kg/m^2^ or higher) is a risk factor for the development of serious chronic diseases, including type 2 diabetes mellitus (T2DM), cardiovascular disease (CVD), non-alcoholic fatty liver disease (NAFLD) and chronic kidney disease [2, 3]. CVD (and indirectly, T2DM) is the leading cause of death and disability due to high BMI [4]. The pathogenesis of obesity is not completely understood. A substantial number of behavioural and lifestyle factors influence susceptibility to obesity; however, the risk conferred by genetic and epigenetic traits is also significant with a heritability of 40–70% [5]. Genome-wide association studies (GWAS) have identified a large number of loci robustly associated with obesity-related traits, such as BMI and body fat percentage (BF%), although the causal genes in these loci remain to be established and characterised.

One such locus identified in GWAS meta-analyses of BF% [6, 7] is near the Sprouty RTK signalling antagonist 2 (*SPRY2*) gene with no other genes within a 1 Mb window around the GWAS signal (Additional file 1: Figure S1). The lead variant (rs534870), 54 kb downstream of *SPRY2*, exhibited a modest association with BMI, body weight and risk of obesity; importantly, its major allele was associated with a 0.14% decrease in BF% in individuals of European descent [6]. In a larger follow-up study, the lead variant (rs693839, in high linkage equilibrium with rs534870, *R*^*2*^ > 0.95) near *SPRY2* was similarly found to have a greater effect on BF% than BMI, suggesting a primary association with adiposity and body fat distribution rather than overall body weight. Additional experiments in *Drosophila* supported *SPRY2* as the likely causal gene [7]. Furthermore, several studies have implicated *SPRY2* as a potential candidate gene for T2DM. The rs1359790 variant [8], situated 193 kb upstream to *SPRY2*, was significantly associated with susceptibility to T2DM in Chinese [9] and Japanese [10] individuals. Sprouty proteins are negative regulators of receptor tyrosine kinase (RTK) signalling pathways [11], which mediate a wide variety of key cellular processes, including critical roles in proliferation, communication and differentiation, as well as influences on motility, metabolism and survival [12]. *SPRY2* specifically modulates the Ras/mitogen activated protein (MAP) kinase pathway [13, 14] and may function as a tumour suppressor gene, since its expression has been found to be repressed in a variety of cancers (reviewed in [15]). Other examples of RTK families include vascular endothelial growth factors (VEGF), insulin-like growth factors (IGF), fibroblast growth-factors (FGF) and platelet-derived growth factors (PDGF).

In a recent study utilising whole-genome RNAi [16], *SPRY2* was identified as a novel regulator of insulin transcription, and deletion of *SPRY2* in adult mouse β-cells led to mild hyperglycaemia and hypoinsulinaemia. However, based on the GWAS findings, there is reason to believe that *SPRY2* may also be involved in peripheral insulin resistance, metabolic syndrome or hepatosteatosis, rather than just insulin secretion. To our knowledge, no prior studies have explored the potential role of *SPRY2* in cells or tissues relevant to these conditions.

The liver is a central metabolic organ and plays a critical role in lipid metabolism and glucose homeostasis. Hence, we aimed to functionally characterise *SPRY2* in HepG2 cells, an in vitro model of human hepatocytes widely studied in the context of glucose and lipid metabolism and insulin resistance [17–19]. We observed a marked increase in glucose uptake, along with an increase in lipid droplet accumulation in HepG2 cells after knockout of *SPRY2*. Transcriptomic profiling revealed differentially expressed genes related to cholesterol biosynthesis, regulation of cell cycle and cellular signalling. These findings suggest a role for *SPRY2* in hepatocyte metabolism and provide further evidence that *SPRY2* is the likely causal gene in a well-established locus associated with body fat distribution and T2DM.

## Methods

### Cell culture

Human hepatoma HepG2 cells (ATCC, HB-8065) were cultured in DMEM + GlutaMAX (Gibco; containing 1 g/L glucose) supplemented with 10% foetal bovine serum (FBS), 100 units/mL penicillin, 0.1 mg/mL streptomycin (all Gibco) and 5 μg/mL plasmocin (Invivogen). Cells were serum-starved overnight prior to assays.

### CRISPR-Cas9 genome editing

Single guide RNAs (sgRNA) targeting two distinct regions of the human *SPRY2* gene were designed using the online tool at: www.broadinstitute.org/gpp/public/analysis-tools/sgrna-design (Additional file 1: Figure S2) and cloned into the BsmBI site of the lentiCRISPRv2 lentiviral vector (Feng Zhang; Addgene #52961) according to [20]. The sgRNA sequences were: 5′-AGTCTCACTGTTGTACACGAtgg-3′ and 5′-GGTTGCCTTAAATTGTGCCAggg-3′ (PAM sequences shown in lower case letters). Correct insertion was verified by Sanger sequencing. Lentiviruses expressing Cas9 and the sgRNA were generated in HEK293T cells by co-transfection of the packaging plasmids psPAX.2 (Didier Trono; Addgene #12260) and psMD2 (David Ron; Addgene #21799). Supernatants containing lentivirus were harvested 24 h and 48 h post-transfection. The pLJM1-EGFP plasmid (David Sabatini; Addgene #19319) was used as a transduction control. HepG2 cells were transduced in OptiMEM (Gibco) containing 8 μg/mL hexadimethrine bromide (polybrene; Sigma-Aldrich) and LentiBlast Reagent (OZ Biosciences). Mock (control) and selection control cells received OptiMEM in place of lentivirus. Transduced cells were selected with puromycin (Gibco) at a concentration of 1 μg/mL for 5–7 days.

### Assessment of CRISPR-Cas9 editing efficiency

Assessment of CRISPR-Cas9 genome editing efficiency for *SPRY2* was determined by sequencing of isolated alleles of the targeted region as described in Additional file 1. Independent transductions were assessed for efficiency of genome editing using Tracking of Indels by Decomposition (TIDE) [21] (Additional file 1: Figure S3). Confirmation of *SPRY2* genome editing was also determined at the protein and mRNA level.

### Overexpression of *SPRY2*

The coding sequence of the *SPRY2* gene was amplified from human cDNA using the following primers: 5′-cc**gctagc**cacc**ATG**GAGGCCAGAGCTCAGAGTGGC-3′ and 5′-cc**ttcgaaCTA**TGTTGGTTTTTCAAAGTTCC-3′ (start and stop codons are shown in bold capital letters; restriction sites used for cloning are shown in bold small letters). Amplification was carried out using Q5 High-Fidelity DNA Polymerase (New England Biolabs) with the following PCR conditions: 30 s at 98 °C, followed by two touchdown cycles of: 7 s at 98 °C, 20 s at 60 °C, 59 °C, 58 °C and 70 s at 72 °C and ending with 26 cycles of: 7 s at 98 °C, 20 s at 57 °C and 70 s at 72 °C and a final extension of 2 min at 72 °C. The expected 968 bp DNA band was isolated, digested with NheI/BstB1 and cloned in the lentiviral vector pLJM1-EGFP that had previously been cut with NheI/BstB1, excising the coding EGFP sequence. The obtained vector (pLJM1-SPRY2) was sequenced before transduction was performed as described above.

### Glucose uptake assay

Glucose uptake was measured using the fluorescent glucose analogue, 2-NBDG (2-(N-(7-Nitrobenz-2-oxa-1,3-diazol-4-yl)Amino)-2-Deoxyglucose; Thermo Fisher) [22]. Cells were cultured and differentiated in black-sided 96-well plates. On the day of the assay, cells were glucose- and serum-starved for 45 min, then 100 μg/mL 2-NBDG in glucose- and serum-free medium was applied for 30 min. Cells were rinsed twice in PBS and a solution of 3.3 μM Hoechst 33342 (Thermo Fisher) in PBS was applied to each well. Cells were imaged with the EVOS FL Auto Imaging System (Life Technologies) on the GFP and DAPI channels at 10x magnification (9–16 images per well). Images were analysed in CellProfiler v. 2.2.0 [23] using an automated pipeline created in-house to subtract background fluorescence, measure GFP intensity and count the number of nuclei in each image. Mean GFP fluorescence per cell, representing glucose uptake, was then calculated.

### Lipid droplet accumulation

The fluorescent neutral lipid dye, Bodipy 493/503 (4,4-Difluoro-1,3,5,7,8-Pentamethyl-4-Bora-3a,4a-Diaza-*s*-Indacene; Molecular Probes), was used to measure the accumulation of lipid droplets in HepG2 cells, as an alternative to Oil Red O [24]. A stock solution of Bodipy 493/503 was prepared in ethanol at a concentration of 1 mg/mL. Cells were treated with 0.5 μg/mL Bodipy 493/503 and 3.3 μM Hoechst 33342 in PBS and imaged with the EVOS FL Auto Imaging System on the GFP and DAPI channels at 10x magnification (9–16 images per well). Images were analysed in CellProfiler as described for the glucose uptake assay.

### Phospho-kinase Array

Considering the established role of *SPRY2* in RTK signalling [11, 25], we hypothesised that key cellular signalling pathways may be affected in these cells. To investigate this, we performed a phospho-kinase array for the simultaneous determination of phosphorylation levels in 43 protein kinases across multiple pathways relevant to metabolism and insulin resistance (including Akt, MAPK, mTOR and Jak/STAT signalling). This was carried out using the Proteome Profiler Human Phospho-Kinase Array (R&D Systems) following the manufacturer’s protocol for LI-COR near-infrared fluorescence detection. Cells were incubated in serum-free medium with or without insulin (100 nM for 10 min [19]) then lysed in Lysis Buffer 6 from the array kit. Protein concentration was determined using the bicinchoninic acid (BCA) assay (Thermo Fisher). Total cellular proteins (300 μg) were hybridised to the array membranes overnight. The arrays were washed and incubated with the detection antibody cocktails for 2 h, followed by IRDye 800CW Streptavidin (LI-COR 925–32,230; 1:2000) for 30 min. Membranes were imaged on the LI-COR Odyssey infrared imaging system and density of spots determined using Fiji [26].

### RNA extraction, library preparation and RNA sequencing (RNA-Seq)

RNA was extracted from mock and *SPRY2* KO HepG2 cells (four preparations each) and sequenced on the Ion Proton System using the Ion PI Hi-Q Sequencing 200 Kit (Thermo Fisher). Full details are provided in Additional file 1.

### Analysis of RNA-Seq data

Differential expression of genes in mock and *SPRY2* KO samples was assessed using the DESeq2 R package from Bioconductor (http://bioconductor.org/packages/release/bioc/html/DESeq2.html). Enrichment of functional terms: gene ontology (GO) molecular process, GO biological process and GO cellular component, PANTHER pathways [27] and Reactome pathways [28] was performed with the AmiGO 2 tool. Analysis methods are described in detail in Additional file 1.

### Quantitative reverse transcription polymerase chain reaction (RT-qPCR)

RNA extraction, reverse transcription and real-time PCR were carried out as outlined in Additional file 1. Primer sequences for genes of interest identified by RNA-Seq and the candidate reference genes *RPL13*, *HPRT* and *B2M* are shown in Additional file 1: Table S1. qPCR data analysis was carried out with qbase+ software, version 3.1 (Biogazelle; www.qbaseplus.com), which included a geNorm reference gene study to determine the most stable reference genes for accurate data normalisation.

### Statistical analysis

All results are presented as mean ± SD. Raw fluorescence data were combined and corrected according to the method described in [29] to eliminate additive and multiplicative systematic errors. Statistical analyses were performed using GraphPad Prism version 5.00 for Windows, GraphPad Software, www.graphpad.com, with *P* < 0.05 considered significant. Details of specific statistical tests are shown in the Figure legends.

## Results

### Confirmation of successful CRISPR-Cas9 genome editing

**Fig. 1:**
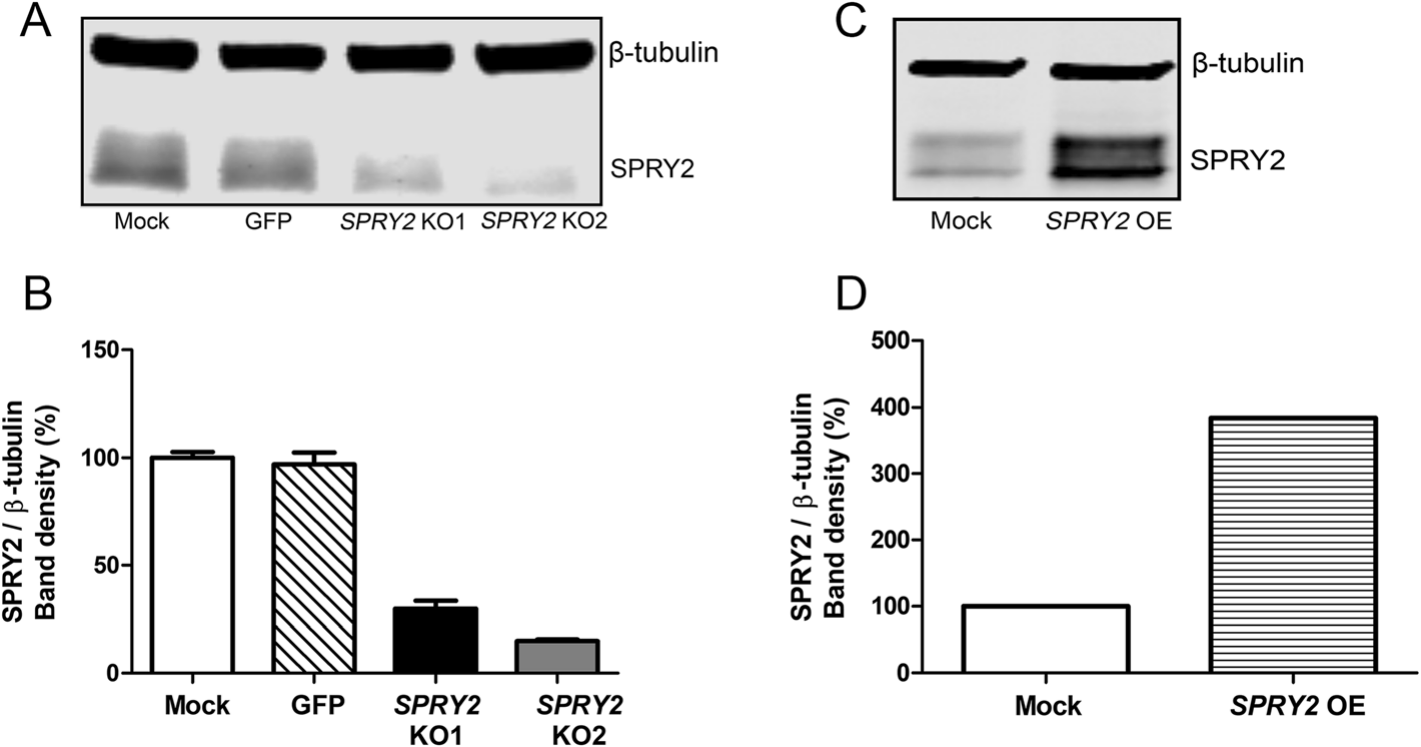
Confirmation of CRISPR-Cas9 genome editing for *SPRY2*. Near-infrared Western blotting was carried out to simultaneously detect SPRY2 and β-tubulin in HepG2 untreated (mock) and CRISPR-Cas9 genome edited (KO/OE) cell lysates. SPRY2 band density was quantified in Fiji, normalised to β-tubulin and expressed as a percentage of mock. **a** Representative Western blot for KO study and **b** band density analysis showing mean + SD of 2 independent transductions. ‘GFP’ denotes transduction control cells expressing GFP using the pLJM1-EGFP lentiviral vector. **c** Western blot for *SPRY2* overexpression (OE) study and **d** corresponding band density analysis. Full-length blots are shown in Additional file 1: Figure S8

For the *SPRY2* KO studies, HepG2 cells were transduced with two distinct CRISPR-Cas9 sgRNA-lentiviral constructs to minimise the risk of off-target effects, which can compromise specificity [30]. The resultant transduced cultures are referred to as *SPRY2* KO1 and *SPRY2* KO2. Western blot analyses showed SPRY2 protein expression to be reduced to 30 ± 4% of mock levels in the *SPRY2* KO1 cells, and 15 ± 1% in the *SPRY2* KO2 cells (mean ± SD of 2 independent transductions; Fig. 1a-b). For the *SPRY2* overexpression (OE) study, transduction of HepG2 cells with the pLJM1-SPRY2 lentiviral vector led to a 4-fold increase in SPRY2 band density compared to mock (Fig. 1c-d).

### *SPRY2* KO increases glucose uptake and lipid droplet accumulation in HepG2 cells

**Fig. 2:**
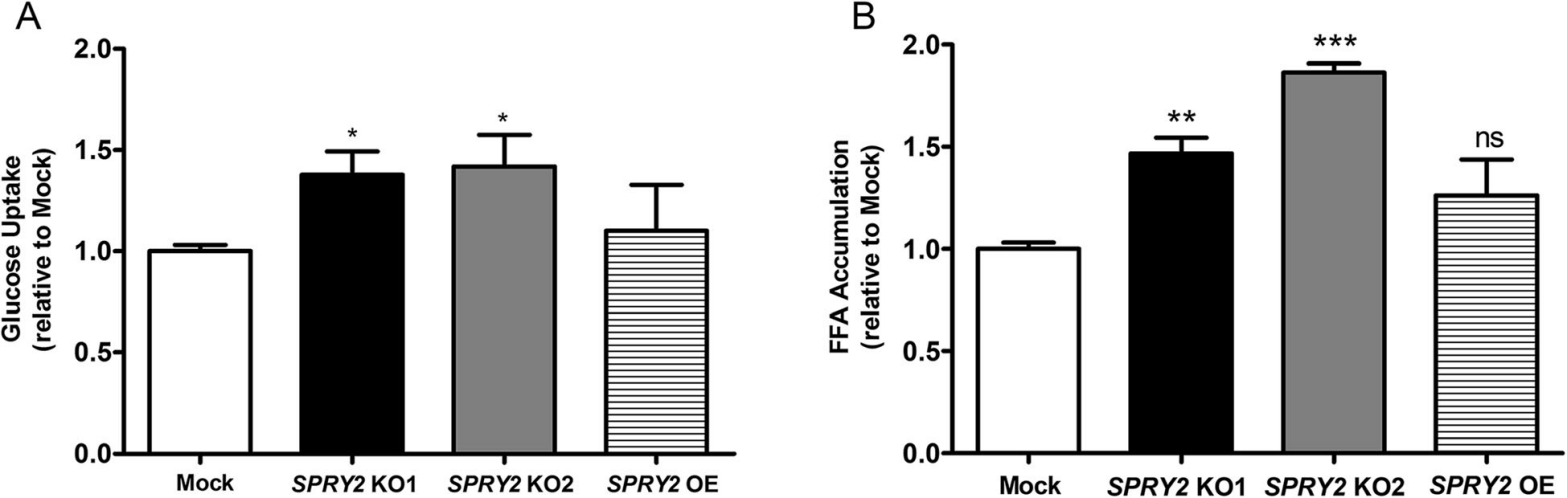
*SPRY2* KO increases glucose uptake and lipid droplet accumulation in HepG2 cells. **a** HepG2 mock, *SPRY2* KO and *SPRY2* OE cells were treated with 100 μg/mL 2-NBDG for 30 min. Cells were washed in PBS, treated with 3.3 μM Hoechst 33342 in PBS and imaged on the GFP and DAPI channels of the EVOS fluorescence microscope. The mean fluorescence per cell, representing glucose uptake, was calculated using CellProfiler and expressed relative to mock. Bars depict mean + SD from 3 independent experiments, with 2 to 4 wells per condition, 9–16 images per well. *P < 0.05 by one-way ANOVA with Bonferroni’s post-hoc test comparing KO with mock. **b** Cells were treated with Bodipy 493/503 (0.5 μg/mL) to label intracellular lipids and imaged using fluorescence microscopy. CellProfiler was used to calculate the mean GFP fluorescence per cell, representing lipid droplet accumulation. Bars depict mean + SD from 3 independent experiments, relative to mock, with 2 to 4 wells per condition, 9–16 images per well. **P < 0.01, ***P < 0.001 by one-way ANOVA with Bonferroni’s post-hoc test; ‘ns’ denotes not significant

The fluorescent glucose analogue, 2-NBDG, was used to measure glucose uptake in HepG2 mock and *SPRY2* KO cells. Knockout of *SPRY2* resulted in significantly higher glucose uptake compared to mock (Fig. 2a; Additional file 1: Figure S4). Glucose uptake was increased by (fold changes relative to mock): 1.4 ± 0.1 in the *SPRY2* KO1 cells and 1.4 ± 0.2 in the *SPRY2* KO2 cells (*P* < 0.05). There were no differences in glucose uptake between HepG2 mock cells and those overexpressing *SPRY2*.

To measure lipid droplet accumulation, the neutral lipid stain, Bodipy 493/503, was applied and visualised with fluorescence microscopy. Lipid droplet accumulation was significantly higher in *SPRY2* KO1 and KO2 cells vs. mock (fold changes relative to mock: 1.5 ± 0.1 for *SPRY2* KO1, *P* < 0.01; 1.9 ± 0.04 for *SPRY2* KO2, *P* < 0.001). There were no differences in lipid droplet accumulation between *SPRY2* OE and mock cells (Fig. 2b; Additional file 1: Figure S5).

### Extent of protein kinase phosphorylation following *SPRY2* KO

Next, the phosphorylation of 43 protein kinases was simultaneously investigated using a phospho-kinase array. Untreated and insulin-stimulated mock and *SPRY2* KO2 cell lysates were evaluated for each cell type; *SPRY2* KO2 cultures were selected since they displayed more compelling phenotypes than *SPRY2* KO1 in the metabolic assays. However, there were no significant changes in any of the 43 phosphorylated protein kinases between mock and KO cells, based on our a priori defined requirement of ≥2.0-fold difference (Additional file 1: Figure S6).

### *SPRY2* KO impacts transcript levels across multiple metabolic pathways

**Fig. 3:**
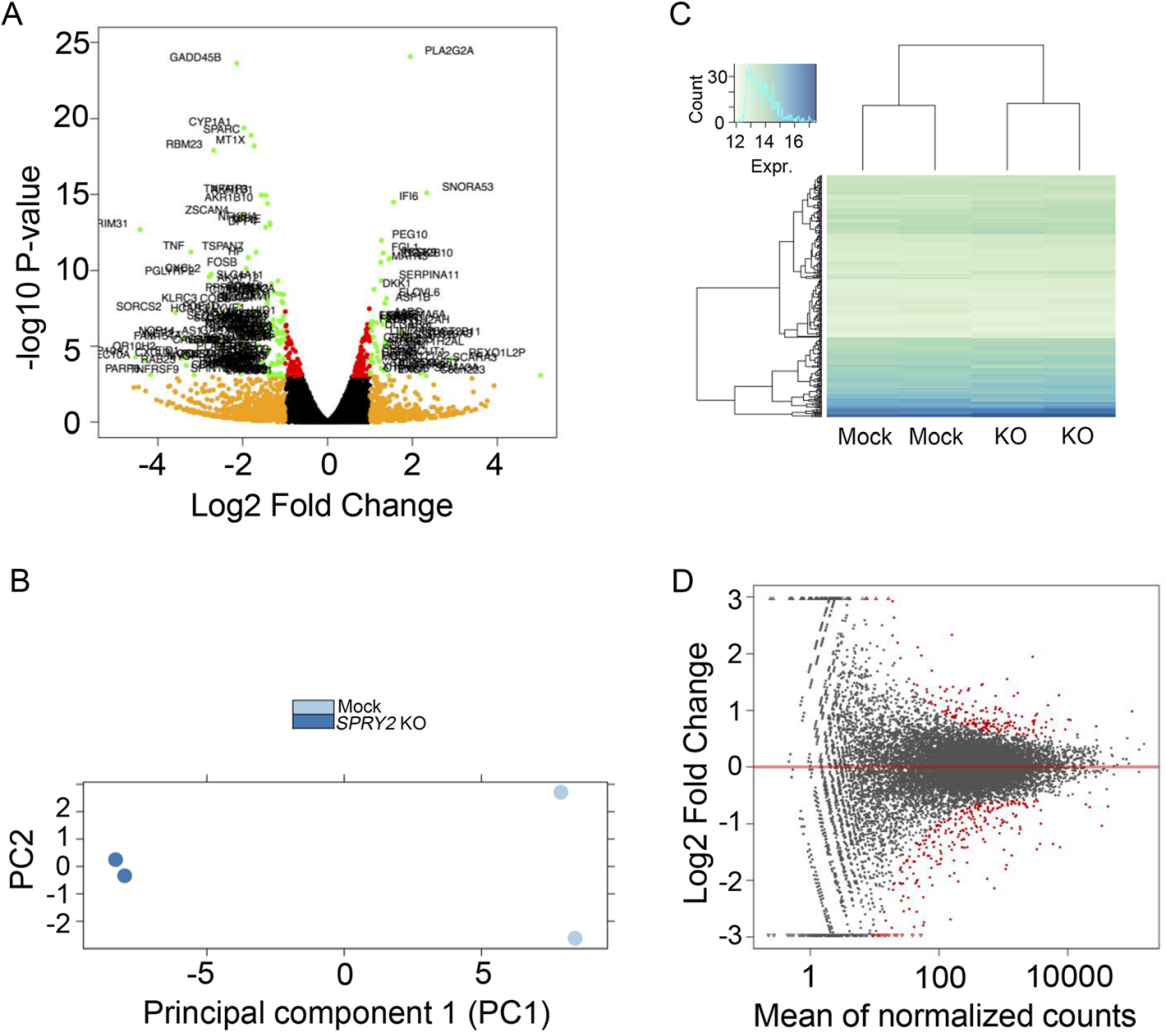
Effect of *SPRY2* KO on transcript levels in HepG2 cells by RNA-Seq. **a** Volcano plot depicting up- and downregulated genes (green) resulting from *SPRY2* KO in HepG2 cells. **b** Principal component analysis (PCA) showing separation of mock and *SPRY2* KO samples along the major PC1 axis. **c** Clustering of the top 100 differentially expressed genes indicating separation of mock and *SPRY2* KO samples. **d** MA plot showing relationship between *P* value and fold change; DE genes are labelled in red

**Fig. 4:**
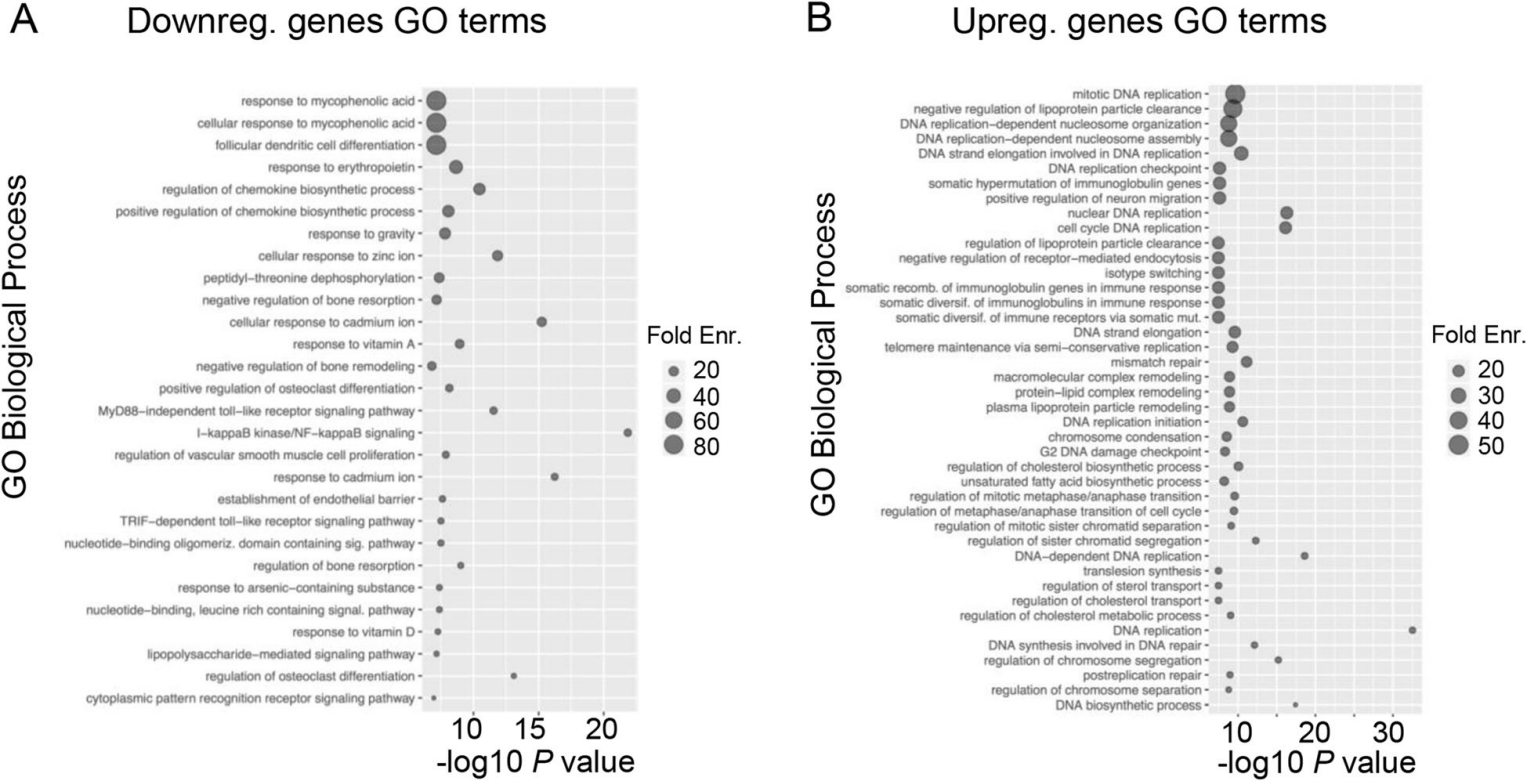
Enrichment analysis of DE genes following *SPRY2* KO in HepG2 cells. Top GO Biological Process terms identified in **a** downregulated and **b** upregulated DE genes from RNA-Seq analysis. The value of -log10 *P* reflects the significance of the GO term enrichment (Enr)

We investigated downstream effects of *SPRY2* KO at the transcriptional level in HepG2 cells using RNA-Seq (Figs. 3 and 4; Additional file 1: Figure S7). Analysis of differential expression using DESeq2 revealed 178 differentially expressed (DE) upregulated genes and 243 DE downregulated genes (FDR < 0.1, fold changes ≥2.0) when comparing *SPRY2* KO2 to mock RNA samples (Fig. 3; Additional file 2).

The DE genes were subsequently tested for the enrichment of GO biological process, GO molecular process, GO cellular component, PANTHER and Reactome pathways in order to classify them by general biological function and pathway involved. Within GO biological processes (Fig. 4), the upregulated DE genes showed enrichment over the expected number of genes per category for mitotic DNA replication (51-fold), cell cycle regulation (G1/S transition: 10-fold, metaphase/anaphase: 13-fold), plasma lipoprotein particle remodelling (17-fold), regulation of cholesterol biosynthetic process (14-fold) and unsaturated fatty acid biosynthetic process (14-fold). In the set of downregulated DE genes, the categories were more diverse and included cellular responses to metal ion (zinc: 22-fold, cadmium: 19-fold, calcium: 6-fold) and regulation of toll-like receptor (TLR) signalling pathways (14-fold), among others. Pathway analysis results are summarised in Additional file 3. Notably, within GO molecular processes, upregulated genes were enriched in low (23-fold) and very low density lipoprotein (VLDL; 90-fold) particle receptor binding. Within the GO cellular component, upregulated genes were enriched for high-density lipoprotein particle (18-fold), while the group of downregulated genes showed 53-fold enrichment of the inhibitor of κB (I-κB)/nuclear factor κB (NF-κB) complex. Upregulated Reactome pathways were mainly related to cell cycle, but also to lipid digestion, mobilisation and transport (7-fold), VLDL interactions (16-fold) and regulation of cholesterol biosynthesis by SREBF (9-fold).

### Validation of RNA-Seq results

The top 20 differentially expressed genes identified by RNA-Seq that were obtained using DE analysis are shown in Table 1. *PLA2G2A* (secretory phospholipase A2 group IIA) was the most significant upregulated gene (fold change 3.9; adjusted *P* = 1.43E^− 20^), which is of particular interest to metabolic diseases in the context of atherogenesis [31], hepatic cholesterol metabolism [32] and insulin sensitivity [33]. The most significant downregulated gene was *GADD45B* (growth arrest and DNA damage inducible beta; fold change 0.2; adjusted *P* = 2.00E^− 20^), which is involved in cellular signalling events related to cell survival, DNA repair and apoptosis [34]. There were several other notable DE genes within the top 20 list with relevance to obesity and T2DM, including: *DPP4* (dipeptidyl peptidase 4) [35], *TNFAIP3* (tumour necrosis factor alpha inducible protein 3), *CYP1A1* (cytochrome P450 family 1 subfamily A member 1) and *AKR1B1* (aldo-keto reductase family 1 member B). Finally, nominal significance was observed for genes involved in glucose transport, fatty acid synthesis and glycolysis, including sterol regulatory element-binding protein 1 (*SREBF1*) and glucokinase regulator (*GCKR*) (Additional file 1: Figure S7).

**Table 1:** Top 20 DE genes in RNA-Seq analysis of HepG2 mock and *SPRY2* KO cells

Symbol	Gene Name	HepG2	
		log2FC	Adjusted *P*
*PLA2G2A*	Phospholipase A2 group IIA	3.86	1.43E-20
*GADD45B*	Growth arrest and DNA damage inducible beta	0.23	2.00E-20
*CYP1A1*	Cytochrome P450 family 1 subfamily A member 1	0.26	2.28E-16
*SPARC*	Secreted protein acidic and cysteine rich	0.29	5.24E-16
*MT1X*	Metallothionein 1X	0.30	2.11E-15
*RBM23*	RNA binding motif protein 23	0.16	3.43E-15
*SNORA53*	Small nucleolar RNA, H/ACA box 53	5.05	1.92E-12
*AKR1B1*	Aldo-Keto reductase family 1 member B	0.37	2.19E-12
*TNFAIP3*	TNF alpha induced protein 3	0.34	2.19E-12
*IFI6*	Interferon alpha inducible protein 6	2.94	5.43E-12
*AKR1B10*	Aldo-Keto reductase family 1 member B10	0.38	6.03E-12
*ZSCAN4*	Zinc finger and SCAN domain containing 4	0.25	3.70E-11
*NFKBIA*	NFKB inhibitor alpha	0.39	9.43E-11
*MT1E*	Metallothionein 1E	0.39	1.22E-10
*DPP4*	Dipeptidyl peptidase 4	0.36	1.66E-10
*TRIM31*	Tripartite motif containing 31	0.05	2.17E-10
*PEG10*	Paternally expressed 10	2.41	1.07E-09
*TNF*	Tumour necrosis factor	0.11	5.71E-09
*TSPAN7*	Tetraspanin 7	0.31	5.75E-09
*FGL1*	Fibrinogen like 1	2.48	6.23E-09

Values are based on the lowest adjusted *P* values and log2 fold changes (log2FC) ≥ 2.0

**Fig. 5:**
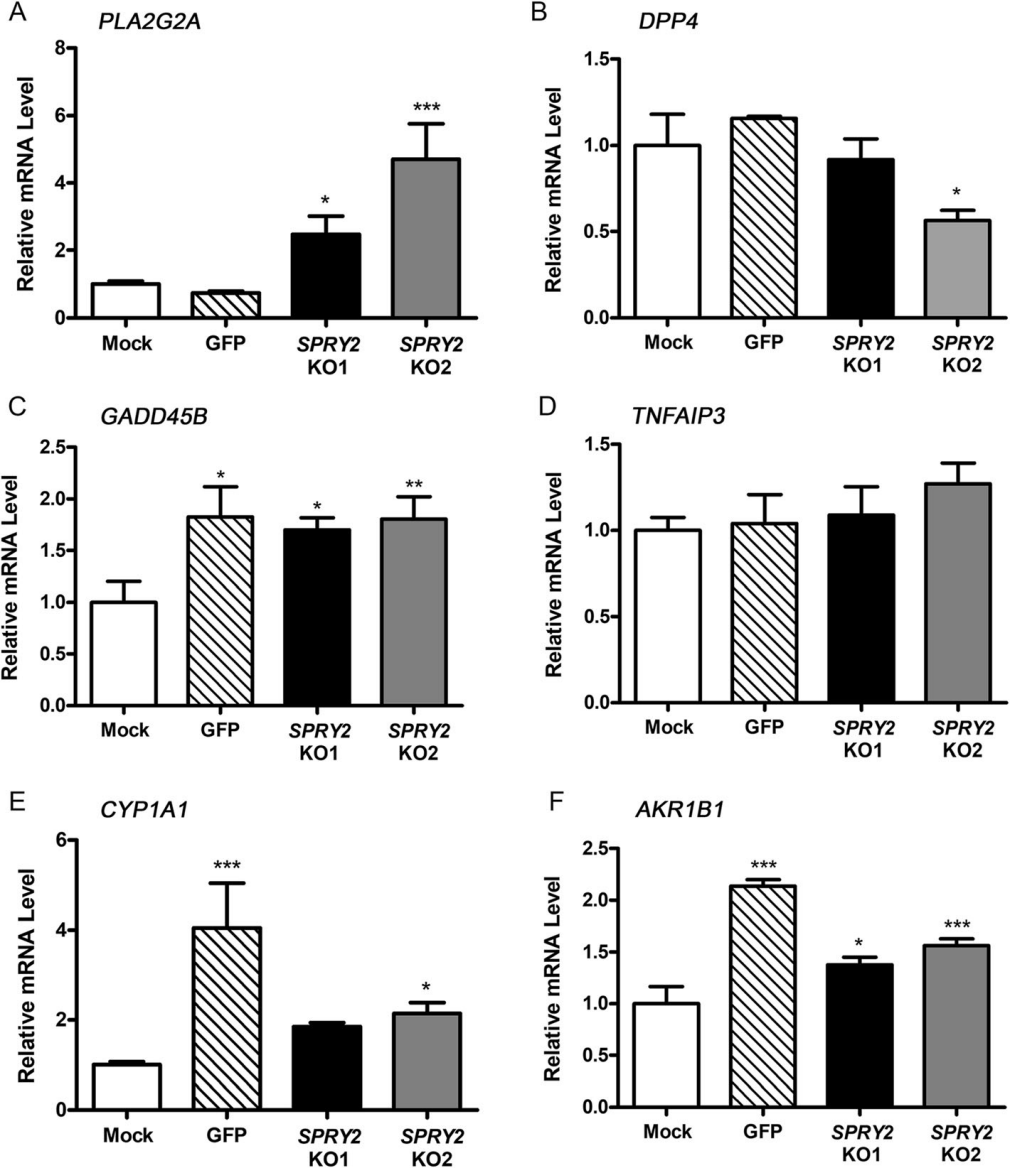
Validation of RNA-Seq results by RT-qPCR. Relative mRNA level in HepG2 mock, GFP and *SPRY2* KO cells as assessed by RT-qPCR, for: **a** *PLA2G2A*, **b** *DPP4*, **c** *GADD45B*, **d** *TNFAIP3*, **e** *CYP1A1* and **f** *AKR1B1*. Data were analysed in qbase+ and normalised to *HPRT* and *RPL13*, determined by geNorm to be the most stable reference genes. Bars show mean + SD from 3 biological replicates each, quantified in triplicate and expressed relative to mock. *P < 0.05, **P < 0.01, ***P < 0.001 by one-way ANOVA with Dunnett’s post-hoc test to compare all bars to mock

We subsequently carried out RT-qPCR experiments to validate selected genes of interest from the top 20 list of DE genes in HepG2 mock, GFP and *SPRY2* KO cells (Fig. 5). *PLA2G2A* mRNA level was confirmed to be significantly upregulated in both *SPRY2* KO1 and KO2 cells (fold changes relative to mock: 2.7 ± 0.5 for *SPRY2* KO1, *P* < 0.05; 4.7 ± 1.1 for *SPRY2* KO2, *P* < 0.001). There was a significant decrease in *DPP4* mRNA level in the *SPRY2* KO2 cells (0.6 ± 0.06 relative to mock, *P* < 0.05). There were no differences in *TNFAIP3* mRNA level between any of the samples. Finally, significant changes were detected in the *SPRY2* KO samples relative to mock (but not GFP) in the following genes: *GADD45B*, *CYP1A1* and *AKR1B1*.

## Discussion

To our knowledge, this is the first study to functionally characterise *SPRY2*, a gene highlighted by GWAS for BF% [6] and T2DM [8], in human hepatocytes. Our results show increased glucose uptake and elevated lipid droplet accumulation to be the major functional consequences of *SPRY2* KO in these cells, as well as modifications to transcript levels of several genes and biological pathways relevant to the pathogenesis of metabolic diseases, such as obesity and T2DM.

The liver is a central metabolic organ and plays a critical role in lipid metabolism and glucose homeostasis. These processes can be dysregulated in obesity, leading to metabolic abnormalities that are associated with the development of NAFLD, insulin resistance and the pathogenesis of T2DM [36]. HepG2 cells are widely used for the study of glucose and lipid metabolism, insulin signalling and mechanisms of insulin resistance in vitro [17–19], and were therefore selected as a model of human hepatocytes in the present study.

The observed significant increase in glucose uptake following *SPRY2* KO in HepG2 cells suggests a possible role for *SPRY2* in glucose metabolism in hepatocytes. However, the mechanism by which this enhanced glucose uptake occurs is unclear; our RNA-Seq analysis revealed a nominal reduction in transcript levels of *SLC2A1* (GLUT1, the major glucose transporter in HepG2 cells [37]) in *SPRY2* KO cells compared to mock.

Given the association of the rs534870 variant near *SPRY2* with BF% [6], as well as its postulated specific effect on adiposity [7], we hypothesised that *SPRY2* KO could affect lipid droplet accumulation in HepG2 cells. Indeed, there were significantly more lipid droplet in the *SPRY2* KO cells compared to mock; this may be a result of de novo fatty acid synthesis due to increased glucose uptake [38]. Our RNA-Seq data showed that *SPRY2* KO led to an increase in transcript level of enzymes involved in lipogenesis, including acetyl-CoA carboxylase alpha (*ACACA*), the rate-limiting step in long-chain fatty acid synthesis [39], as well as fatty acid synthase (*FASN*). Furthermore, *SPRY2* KO may enhance the glycolytic pathway that provides pyruvate for fatty acid synthesis: our RNA-Seq results showed decreased transcript levels of *GCKR*, which negatively regulates hepatic glucokinase (GCK), a glycolytic enzyme that phosphorylates glucose to produce glucose-6-phosphate [40], as well as a significant increase in transcript level of *SREBF1*, a transcription factor that mediates insulin-stimulated upregulation of GCK [41]. Therefore, *SPRY2* KO may affect glucose uptake and lipid droplet accumulation in part by modulating activation and expression of insulin-sensitive GCK enzymes, although these observations require validation in future studies.

A clear phenotype resulting from *SPRY2* OE in HepG2 cells did not emerge in the present study, with similar levels of glucose uptake and lipid droplet accumulation observed in *SPRY2* OE and mock cells. Gain- and loss-of-function studies of a target gene are generally expected to produce opposite phenotypes, however, this is not always observed [42, 43], particularly with regard to tumour suppressor genes [44], which may apply in the case of *SPRY2*. Ideally, the *SPRY2* OE experiments would be repeated to further characterise the resultant phenotypes in HepG2 cells.

Further investigation into cellular signalling pathways via phospho-kinase profiling did not reveal any significant changes to protein kinase phosphorylation levels between HepG2 mock and *SPRY2* KO cells, despite the array encompassing a number of protein kinases relevant to glucose metabolism and insulin signalling. Transcriptome profiling via RNA-Seq identified a total of 421 DE genes between mock and *SPRY2* KO HepG2 cells (178 upregulated and 243 downregulated genes). The most significant DE gene, *PLA2G2A*, was validated by RT-qPCR as significantly upregulated at the mRNA level following *SPRY2* KO. *PLA2G2A* is highly expressed in liver and elevated circulating levels of its encoded protein, secretory phospholipase A2 group IIA (sPLA_2_), are a risk factor for atherogenesis [31]. Furthermore, sPLA_2_ influences hepatic cholesterol uptake [32] and improves insulin sensitivity in mice [33]. Dipeptidyl peptidase 4 (*DPP4*) mRNA level was validated as significantly decreased in the *SPRY2* KO2 cells. Elevated DPP4 expression has been linked to insulin resistance in obesity [45] and NAFLD [35], and DPP4 inhibitors are currently in clinical use as anti-diabetic drugs. Further studies will be required to explore the potential link between *SPRY2* and these genes in the context of obesity and T2DM, and in particular, the potential role of *SPRY2* in the pathogenesis of liver diseases, such as NAFLD.

Pathway analysis revealed many DE upregulated genes arising from *SPRY2* KO to be involved with DNA replication and cell cycle regulation; this is consistent with the established role for *SPRY2* in inhibiting cell proliferation and acting as a tumour suppressor in certain types of cancer [14, 46]. Among the highly significant terms several were related to the metabolic processes that regulate cholesterol biosynthesis and fatty acid metabolism, which correlates with our experimental findings suggesting that *SPRY2* regulates metabolic processes, such as glucose uptake and lipid accumulation.

Collectively, the increase in glucose uptake and lipid droplet accumulation, as well as the modulation of transcript levels in *SPRY2* KO cells suggest that *SPRY2* may be involved in metabolic homeostasis in hepatocytes, although whether this contributes to the pathogenesis of obesity, T2DM or NAFLD remains to be determined using further in vitro experiments. However, it would be interesting to speculate that the changes in transcript levels of multiple genes associated with fatty acid synthesis and alterations to cholesterol synthesis pathways observed in the *SPRY2* KO cells could constitute a potential mechanism leading to increased lipid droplet accumulation in hepatocytes, and potentially, a contribution by *SPRY2* to conditions such as hepatic steatosis and NAFLD. Furthermore, the present study is unable to disentangle if the numerous DE cell cycle regulation genes identified in the RNA-Seq experiments are relevant to obesity and T2DM or may also be indicative of neoplastic changes, given that the loss of *SPRY2* is associated with hepatocarcinogenesis [47–49] and that HepG2 cells themselves are a hepatoma cell line.

## Conclusions

We have shown that *SPRY2* KO increases glucose uptake and lipid droplet accumulation in HepG2 cells, and leads to downstream transcriptomic changes in genes relevant to metabolic diseases. Although the present study does not identify the exact molecular mechanisms underlying the observed metabolic changes in the *SPRY2* KO cells, it provides important insights into the function of *SPRY2* in human hepatocytes and contributes to elucidating the potential role of *SPRY2* in the pathogenesis of obesity and T2DM.

## Supplementary information

**Additional file 1: **Supplementary Methods. **Figure S1.** Overview of the *SPRY2* locus. **Figure S2.** Exon organisation of *SPRY2* gene. **Figure S3.** Examples of DNA mutations generated following CRISPR-Cas9 genome editing for *SPRY2* in HepG2 cells. **Figure S4.** *SPRY2* KO increases glucose uptake in HepG2 cells. **Figure S5.** Increased lipid droplet accumulation following *SPRY2* KO in HepG2 cells. **Figure S6.** *SPRY2* KO does not alter protein kinase phosphorylation in HepG2 cells. **Figure S7.** Alterations in the expression of key genes involved in glucose transport, lipogenesis and glycolysis following *SPRY2* KO in HepG2 cells. **Figure S8.** Confirmation of CRISPR-Cas9 genome editing for *SPRY2*. **Table S1.** Primer sequences for RT-qPCR.

**Additional file 2. **Differential gene expression comparing HepG2 mock and *SPRY2* KO cells. Separate tabs for complete list, significantly up- and downregulated transcripts.

**Additional file 3.** Pathway analysis of differentially expressed genes identified by RNA-Seq displaying results for enrichment of PANTHER, Reactome and GO biological process sets.

## Data Availability

The data generated or analysed during this study are included in this published article (and its supplementary information files).

## Abbreviations

BF%: Body fat percentage
BMI: Body mass index
DE: Differentially expressed
GWAS: Genome-wide association studies
KO: Knockout
NAFLD: Non-alcoholic fatty liver disease
RTK: Receptor tyrosine kinase
sgRNA: single guide RNA
SPRY2: Sprouty RTK signalling antagonist 2
T2DM: Type 2 diabetes mellitus
