# Supplementary material for: CRISPR-Cas9-mediated knockout of SPRY2 in human hepatocytes leads to increased glucose uptake and lipid droplet accumulation
Source: BMC Endocr Disord. 2019 Oct 29;19:115. doi: 10.1186/s12902-019-0442-8 (PMC6820957; doi:10.1186/s12902-019-0442-8)
Supplement: Supplementary file 1 — Additional file 1: Supplementary Methods. Figure S1. Overview of the SPRY2 locus. Figure S2. Exon organisation of SPRY2 gene. Figure S3. Examples of DNA mutations generated following CRISPR-Cas9 genome editing for SPRY2 in HepG2 cells. Figure S4. SPRY2 KO increases glucose uptake in HepG2 cells. Figure S5. Increased lipid droplet accumulation following SPRY2 KO in HepG2 cells. Figure S6. SPRY2 KO does not alter protein kinase phosphorylation in HepG2 cells. Figure S7. Alterations in the expression of key genes involved in glucose transport, lipogenesis and glycolysis following SPRY2 KO in HepG2 cells. Figure S8. Confirmation of CRISPR-Cas9 genome editing for SPRY2. Table S1. Primer sequences for RT-qPCR. [file 12902_2019_442_MOESM1_ESM.docx]

**Supplementary Information**

**Additional File 1**

**CRISPR-Cas9-mediated knockout of *SPRY2* in human hepatocytes leads to increased glucose uptake and lipid droplet accumulation**

Naomi L. Cook, Milos Pjanic, Andrew G. Emmerich, Abhiram S. Rao, Susanne Hetty, Joshua W. Knowles, Thomas Quertermous, Casimiro Castillejo-López, Erik Ingelsson

**Supplementary Methods**

**Assessment of CRISPR-Cas9 Editing Efficiency**

Cells resistant to puromycin were propagated and an aliquot corresponding to ∼1 × 10^5^ cells was withdrawn for genomic DNA (gDNA) preparation. PCR amplification of the target sequence was carried out with 20 ng gDNA using Platinum Taq polymerase (Thermo Fisher) and the primers: 5´-GACAAGCAGTGCCTTTGCTC-3´ and 5´-GGTTTTTCAAAGTTCCTAGGGGG-3´. PCR conditions were: 3 min at 94 °C, followed by two touchdown cycles of 30 s at 94 °C, 20 s at 59 °C, 58 °C, 57 °C and 30 s at 72 °C and ending with 27 cycles of 30 s at 94 °C, 20 s at 56 °C and 30 s at 72 °C. The PCR products were purified using the PCR DNA and Gel Band purification kit (GE Healthcare) and cloned using the TOPO TA Cloning kit in the pCR2.1 vector (Thermo Fisher). Individual bacterial clones were Sanger sequenced using universal primers included in the vector. Sequences were aligned to a wild type clone and the frequency of editing was estimated. Independent transductions were assessed for efficiency of genome editing using Tracking of Indels by Decomposition (TIDE) ([1](#_ENREF_1)). PCR products were purified using the SequalPrep Normalisation Plate (Thermo Fisher) and aliquots of 30 ng DNA were Sanger sequenced in both directions using the amplification primers. The chromatograms were subsequently analysed using the online tools at <https://tide.nki.nl/> (Figure S3).

**Western Blotting**

To confirm successful CRISPR-Cas9 genome editing for *SPRY2*, HepG2 cells were lysed in CelLytic M (Sigma-Aldrich) and protein concentrations determined using the BCA assay. Cellular proteins (15-30 µg) were separated on 4-15 % SDS-PAGE gels and transferred to nitrocellulose membranes using the Turbo Transfer system (all Bio-Rad). Primary antibodies were applied overnight in 5% (w/v) fat-free milk powder (Fisher Scientific) in Tris-buffered saline with 0.1% (v/v) Tween-20 (TBST; Sigma-Aldrich). The primary antibody for SPRY2 was from Cell Signaling (#14954; rabbit mAb; 35 kDa; diluted to 1:1000); the primary antibody for β-tubulin was from Sigma-Aldrich (#T4026; mouse mAb; 55 kDa; diluted to 1:2000). LI-COR near-infrared fluorescent secondary antibodies (IRDye 800CW Donkey anti-rabbit IgG (925-32213) and IRDye 680LT Donkey anti-mouse IgG (925-68022); 1:20,000) were applied for 1 h in 5% (w/v) fat-free milk powder in TBST. Blots were visualised using the LI-COR Odyssey infrared imaging system to simultaneously detect SPRY2 and the loading control, β-tubulin. Band density analysis was subsequently carried out using Fiji. Full-length blots are shown in Figure S8.

**RNA extraction, library preparation and RNA sequencing**

RNA was extracted from HepG2 cells (four preparations each of mock and *SPRY2* KO) using the RNeasy Mini Kit (Qiagen), incorporating an on-column DNase treatment (RNase-free DNase set; Qiagen). The concentration and integrity of the RNA was assessed using the Agilent Bioanalyzer RNA 6000 Nano Kit, and RNA purity was measured on the Nanodrop 2000 (Thermo Fisher). RNA sequencing (RNA-Seq) of the HepG2 cells was carried out at the National Genomics Infrastructure platform at the Science for Life Laboratory, Uppsala, Sweden. Reverse transcription of 50 ng each RNA sample was carried out according to the Ion AmpliSeq Transcriptome Human Gene Expression Kit Preparation protocol (Thermo Fisher). The synthesised cDNA was amplified using the Ion AmpliSeq Transcriptome Human Gene Expression core panel (Thermo Fisher). Primer sequences were partially digested and adaptors (Ion P1 Adapter and Ion Xpress Barcode Adapter; Life Technologies) were ligated to the amplicons. Adaptor-ligated amplicons were purified using Agencourt AMPure XP reagent (Beckman Coulter), eluted in amplification mix (Platinum PCR SuperMix High Fidelity and Library Amplification Primer Mix; Thermo Fisher) and amplified. Amplicons were subject to size-selection and purification and quantified using the Fragment Analyzer instrument with the DNF-474 High Sensitivity NGS Fragment Analysis Kit (Advanced Analytical Technologies, Inc.). Samples were subsequently pooled, followed by emulsion PCR on the Ion OneTouch 2 System using the Ion PI Hi-Q OT2 Kit (Thermo Fisher). Finally, samples were loaded onto an Ion PI v3 chip and sequenced on the Ion Proton System using the Ion PI Hi-Q Sequencing 200 Kit (Thermo Fisher). RNA-Seq reads were quality-controlled and analysed using Torrent Suite Software (Thermo Fisher) with standard settings.

**Analysis of RNA-Seq data**

Differential expression of exons, genes and transcripts were assayed using the DESeq2 R package from Bioconductor (<http://bioconductor.org/packages/release/bioc/html/DESeq2.html>), which uses negative binomial distribution to estimate dispersion and model differential expression such as to permit biological variability to be different among tested genes (transcripts). Adjusted *P* values were computed using p.adjust() in R v3.4.0. Enrichment of functional terms: PANTHER pathways, gene ontology (GO) molecular process, GO biological process, GO cellular component and Reactome pathways was performed with the AmiGO 2 tool using Fisher’s Exact test with FDR multiple test correction. In addition, GOseq was used for GO biological process terms enrichment. Principal component analysis (PCA) and hierarchical clustering were performed using standard R functions, plotPCA and heatmap.2, through the RNASeqFPro workflow (<https://github.com/milospjanic/rnaSeqFPro>). However, it was evident from the PCA that good separation of the four mock and four *SPRY2* KO2 samples was not obtained, suggesting the presence of a batch effect or other confounding variable. We initially considered the possibility that the lack of complete clustering of the mock and KO HepG2 samples could be indicative of unsuccessful genome editing; however, Western blotting clearly showed downregulation of the SPRY2 protein (Fig. 1) and gene sequencing demonstrated mutations to the *SPRY2* gene (Figure S3). Hence, the RNA-Seq data were re-analysed to include only the well-separated HepG2 samples (two mock vs. two *SPRY2* KO), and all downstream analyses were performed on these samples only.

**Quantitative Reverse Transcription Polymerase Chain Reaction (RT-qPCR)**

Cellular RNA was extracted from HepG2 cells as described in the ‘RNA Extraction’ section, and cDNA synthesised from 1-2 µg RNA with the High Capacity RT kit (Applied Biosystems) or the QuantiTect Reverse Transcription Kit (Qiagen). Primer sequences for *SPRY2* (flanking the sgRNA target site in exon 2), selected genes of interest identified by RNA-Seq and the candidate reference genes *RPL13*, *HPRT* and *B2M* (Table S1) were designed using Primer-BLAST ([2](#_ENREF_2)) or obtained from the PrimerBank database ([3](#_ENREF_3)) and purchased from Integrated DNA Technologies. Real-time PCR was carried out on the Applied Biosystems StepOne Plus machine using PowerUP SYBR Green master mix (Applied Biosystems) with 300 nM forward and reverse primers and the following conditions: 2 min at 50 °C (UDG activation), 2 min at 95 °C, then 40 cycles of: 15 sec at 95 °C, 15 sec at 60 °C and 1 min at 72 °C. Melt curve analysis was subsequently carried out by slowly heating samples from 65 °C to 95 °C and collecting fluorescence measurements at ~0.7 °C increments. A standard curve (prepared from pooled cDNA) was included for each set of primers and all primer pairs had efficiencies between 90-110% and R^2^ > 0.99. qPCR data analysis was carried out with qbase+ software, version 3.1, which included a geNorm reference gene study to identify the most stable reference genes and optimal number of reference genes required for accurate data normalisation (determined to be *HPRT* and *RPL13*).

**References**

1. Brinkman EK, Chen T, Amendola M, van Steensel B. Easy quantitative assessment of genome editing by sequence trace decomposition. Nucleic Acids Res. 2014;42(22):e168-e.

2. Ye J, Coulouris G, Zaretskaya I, Cutcutache I, Rozen S, Madden TL. Primer-BLAST: a tool to design target-specific primers for polymerase chain reaction. BMC Bioinformatics. 2012;13:134.

3. Wang XW, Spandidos A, Wang HJ, Seed B. PrimerBank: a PCR primer database for quantitative gene expression analysis, 2012 update. Nucleic Acids Res. 2012;40(D1):D1144-D9.

**Supplementary Figures & Table**


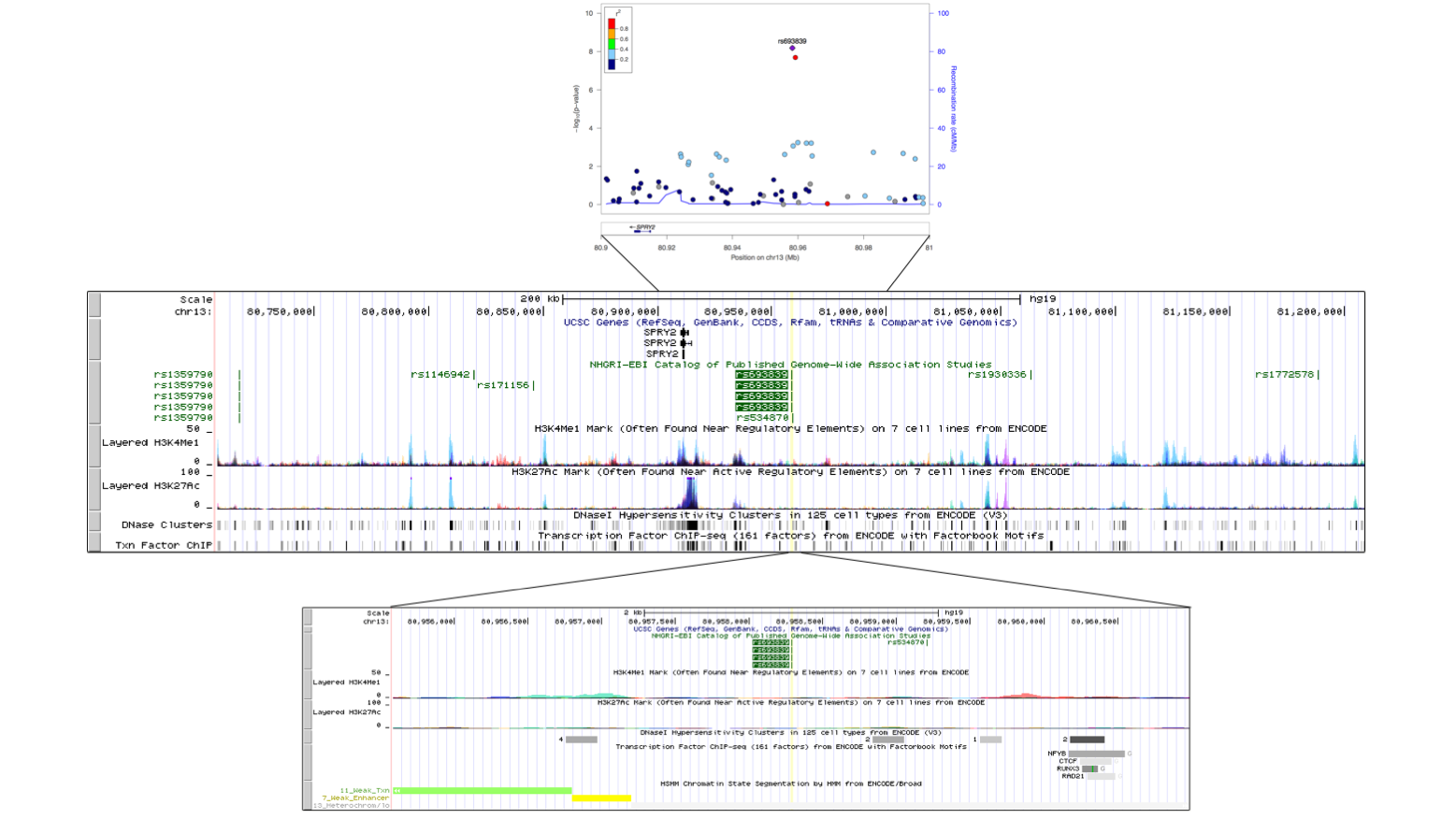


**Figure S1. Overview of the *SPRY2* locus.** Top panel: Locus Zoom plot from the latest GWAS of body fat % (BF%). Middle panel: Screenshot from UCSC genome browser showing the larger region with tracks for genes, GWAS catalogue, enhancer marks and transcription factors. Bottom panel: Close-up of the region just around the BF% GWAS signal with the same regulatory tracks and HMM chromatin state predicting regulatory elements in the region close to the lead variant. The linkage disequilibrium is perfect between the two previously reported lead variants from the two GWAS (D’=1; r^2^=1). There are no eQTLs in the region in relevant tissues.


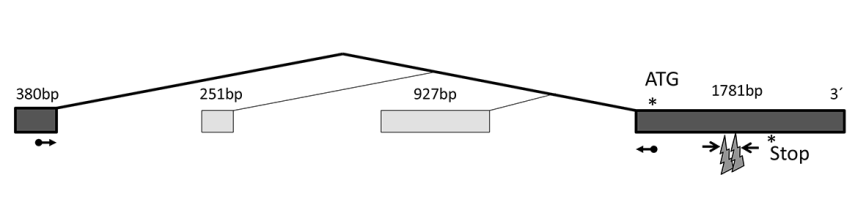


**Figure S2. Exon organisation of *SPRY2* gene.** Exons are showed as boxes, with the length in base pairs above. Major and rare exons are dark and light coloured, respectively. The CRISPR-Cas9 targets are represented by lightning symbols. The start codon (ATG) and the Stop codon are indicated by (*). The primers used to detect the induced mutations are indicated by arrows and the primers used for AmpliSeq (transcriptome profiling) are indicated by a dot followed by an arrow.

**
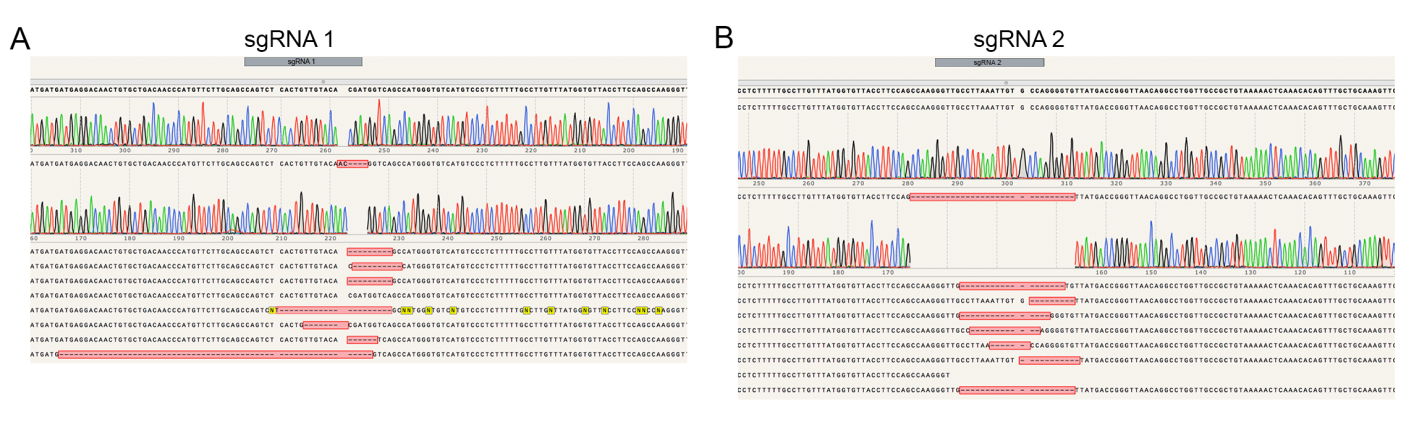
**

**Figure S3. Examples of DNA mutations generated following CRISPR-Cas9 genome editing for *SPRY2* in HepG2 cells.** Chromatograms showing DNA sequences from mock and CRISPR-Cas9 edited cells. Two distinct sgRNA were used to target exon 2 (1,781 bp) of *SPRY2*, just downstream of the translation start site (Note: two new and probably rare exons of 251 bp and 927 bp have recently been annotated upstream of exon 2; these may act as alternative transcription start sites. The longer new exon codes in the inverse direction for a 170 amino acid polypeptide similar to the uncharacterised protein LOC1046661266 from *Rhinopithecus roxellana*). The top chromatogram in each panel represents sequencing of mock cells, while the bottom chromatograms represent isolated alleles, with nucleotide sequences underneath (deletion mutations are highlighted in pink and ambiguous peaks labelled by a yellow ‘N’). The sgRNAs are indicated by the grey boxes above the mock sequences.


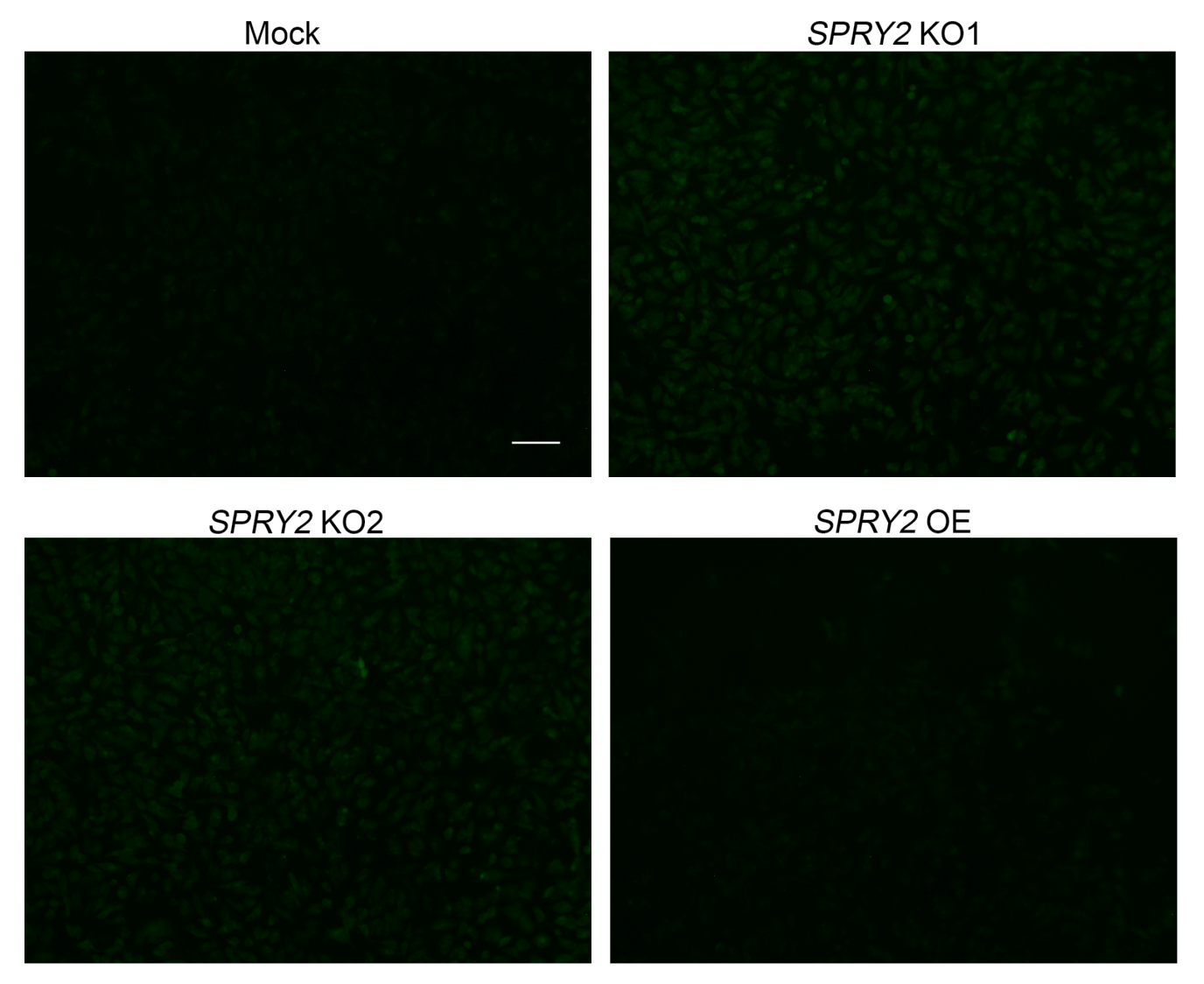


**Figure S4. *SPRY2* KO increases glucose uptake in HepG2 cells.** HepG2 mock, *SPRY2* KO and *SPRY2* OE cells were treated with the fluorescent glucose analogue, 2-NBDG (100 µg/mL) for 30 min and imaged on the GFP channel of the EVOS fluorescence microscope. Glucose uptake was determined as described in the Methods section. Representative 10x GFP images from 3 independent experiments are shown; scale bar = 100 µm.

**
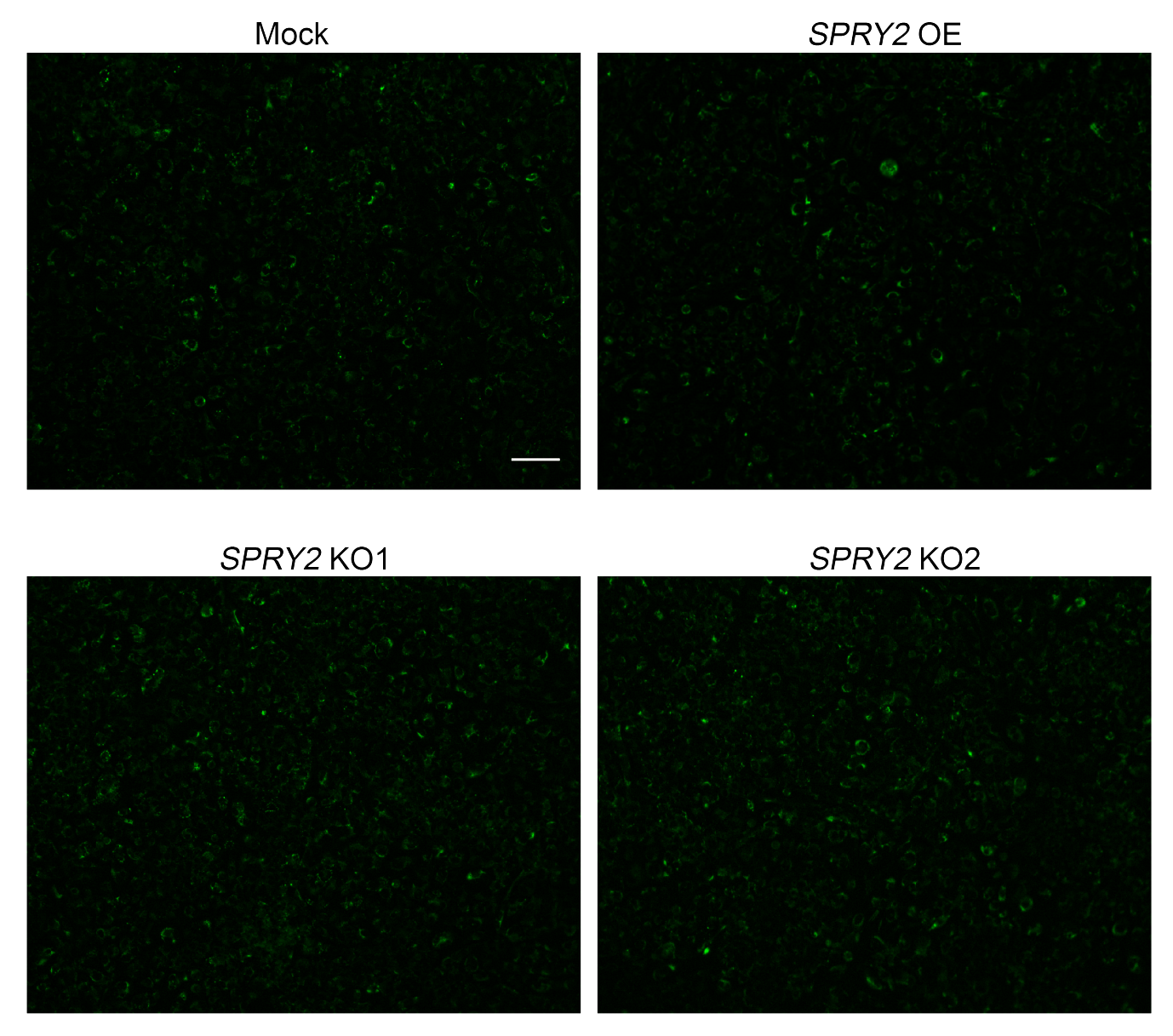
**

**Figure S5. Increased LD accumulation following *SPRY2* KO in HepG2 cells.** HepG2 mock, *SPRY2* KO and *SPRY2* OE cells were treated with the intracellular lipid stain, Bodipy 493/503 (0.5 µg/mL), and imaged on the GFP channel of the EVOS fluorescence microscope. Representative 10x GFP images from 3 independent experiments are shown; background of each image was subtracted in Fiji using a 50 pixel rolling ball radius. Scale bar = 100 µm.

**
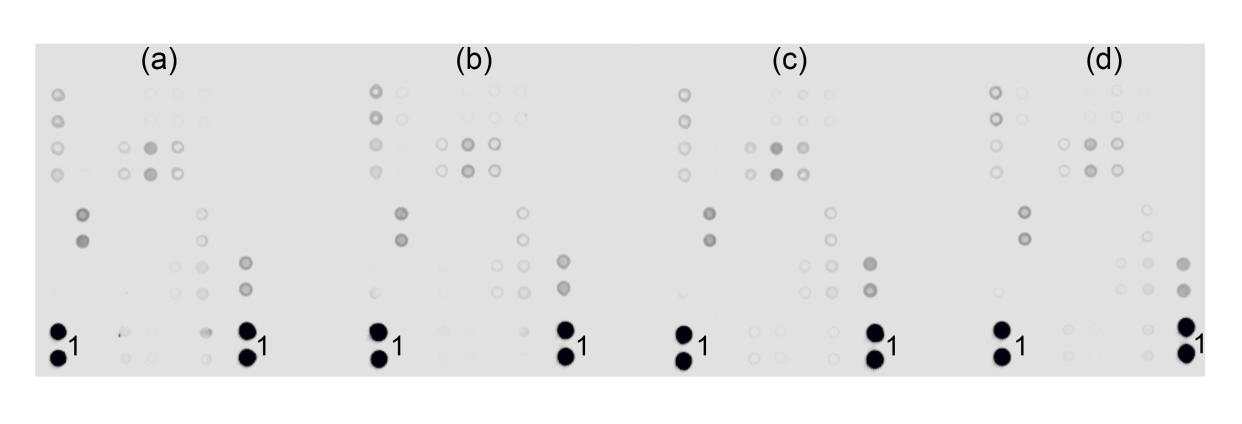
**

**Figure S6.** ***SPRY2* KO does not alter protein kinase phosphorylation in HepG2 cells.** Phospho-kinase arrays to detect phosphorylated proteins in HepG2 cell lysates: (a) mock basal, (b) mock + insulin, (c) *SPRY2* KO2 basal, (d) *SPRY2* KO2 + insulin (100 nM insulin for 10 min). Cells were lysed and 300 µg total cellular proteins were hybridised to the array membranes overnight. The arrays were washed and incubated with the manufacturer’s detection antibody cocktails for 2 h, washed again and incubated with near-infrared IRDye 800CW Streptavidin for 30 min. Membranes were imaged on the LI-COR Odyssey infrared imaging system and density of duplicate spots determined using Fiji. Figure shows Part A membranes; part B not shown. Pairs of spots marked with ‘1’ indicate positive controls.


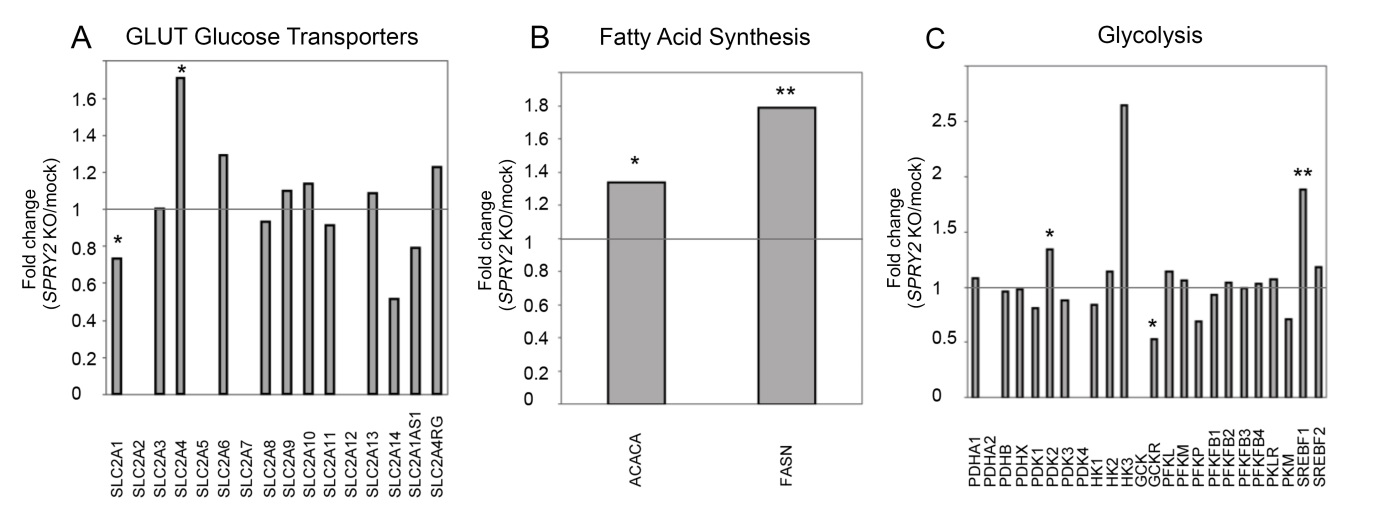


**Figure S7. Alterations in the expression of key genes involved in glucose transport, lipogenesis and glycolysis following *SPRY2* KO in HepG2 cells.** RNA-Seq transcript profiling of (A) the SLC2 (GLUT) family of glucose transporters, (B) the major lipolysis enzymes, acetyl-CoA carboxylase alpha (*ACACA*) and fatty acid synthase (*FASN*) and (C) major control genes of the glycolytic pathway, glucokinase regulator (*GCKR*) and sterol regulatory element binding transcription factor 1 (*SREBF1*). * denotes nominal *P* value significance, ** adjusted *P* value significance.





SPRY2

β-tubulin

37 kDa Marker

Mock, GFP, *SPRY2* KO1, *SPRY2* KO2 (i.e. as per layout in Fig. 1A)

**A**

**B**





β-tubulin

SPRY2

Mock, *SPRY2* OE

(i.e. as per layout in Fig. 1C)

Samples from a

different study

37 kDa Marker

**Figure S8. Confirmation of CRISPR-Cas9 genome editing for *SPRY2*.** Near-infrared Western blotting was carried out to simultaneously detect SPRY2 and β-tubulin in HepG2 untreated (mock) and CRISPR-Cas9 genome edited (KO/OE) cell lysates. SPRY2 band density was quantified in Fiji and normalised to β-tubulin. Representative full-length Western blots for HepG2 (A) KO study and (B) OE study; supplement to Fig. 1.

**Table S1:** Primer sequences for RT-qPCR.

| Gene symbol | Forward Primer (5’→3’) | Reverse Primer (5’→3’) | Amplicon size (bp) |
| --- | --- | --- | --- |
| *HPRT* | tgaggatttggaaagggtgt | tcccatctccttcatcacatc | 88 |
| *RPL13* | agatggcggaggtgcag | gttgatgccttcacagcgta | 128 |
| *B2M* | ggctatccagcgtactccaa | aatgtcggatggatgaaacc | 109 |
| *PLA2G2A* | ATGAAGACCCTCCTACTGTTGG | GCTTCCTTTCCTGTCGTCAACT | 110 |
| *GADD45B* | TACGAGTCGGCCAAGTTGATG | GGATGAGCGTGAAGTGGATTT | 115 |
| *AKR1B1* | GCTTTAAGCCTGGGAAGGAATTT | GCCCACGTGTCCAGAATGTTG | 85 |
| *TNFAIP3* | TTGTCCTCAGTTTCGGGAGAT | ACTTCTCGACACCAGTTGAGTT | 99 |
| *DPP4* | TACAAAAGTGACATGCCTCAGTT | TGTGTAGAGTATAGAGGGGCAGA | 134 |
| *CYP1A1* | ACATGCTGACCCTGGGAAAG | GGTGTGGAGCCAATTCGGAT | 94 |
